# Role of Ancillary Procedures in Facial Rejuvenation

**DOI:** 10.1097/GOX.0000000000002075

**Published:** 2019-06-14

**Authors:** Rod. J. Rohrich, Raja Mohan

**Affiliations:** From the Dallas Plastic Surgery Institute, Dallas, Texas, USA.

## Abstract

Face-lift techniques have evolved beyond the concept of lifting and now include incorporating superficial and deep filling of facial fat compartments to address volume deflation and aging. In addition to traditional face-lift methods involving the excision and tightening of skin and the underlying soft tissues, the following ancillary procedures have become paramount in achieving an optimal result: blending the lid-cheek junction; chin augmentation; lobule augmentation; hand rejuvenation; perioral rejuvenation; chemical peels; and laser resurfacing. We presented a summary of these integral techniques.

## Abstract 2

Supplemental Digital Content is available in the text.

## INTRODUCTION

Face-lift techniques have evolved beyond the concept of lifting and now include incorporating superficial and deep filling of facial fat compartments to address volume deflation and aging.^1,2^ In addition to traditional face-lift methods involving the excision and tightening of skin and the underlying soft tissues,^3^ ancillary procedures have become paramount in achieving an optimal result.^4^ Prior studies have shown no difference in aesthetic outcome with respect to face-lift technique,^5^ but autologous fat grafting and ancillary procedures in facial rejuvenation are performed routinely and safely to address signs of aging and achieve a better aesthetic outcome.^6–11^

**Fig. 1.**
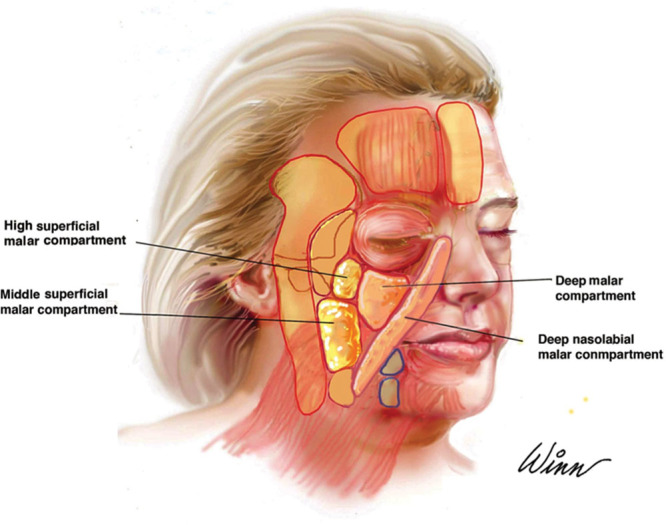
Key fat compartments in lift-and-fill face-lift. Published with permission from *Plast Reconstr Surg.* 2014;133:756e–767e.

The fat compartments of the face have been elucidated by Rohrich^12–14^ and serve as a guide for the regions that require augmentation with autologous fat grafting (Fig. 1). These seminal studies highlighted that the superficial and deep compartments of the face are partitioned by fascial layers.^15^ A large percentage of the grafted autologous fat survives with the presence of autologous stem cells and immediate revascularization.^16^ Some portions are replaced by fibrous tissue.^17^ The goal in autologous fat grafting when used for ancillary procedures is to blend the lower eyelid-cheek junction to achieve an ogee curve.^18^ This procedure can be performed concomitantly with a lower lid blepharoplasty. Volumization of regions that are deflated or atrophic, such as the forehead and periorbitum, also aid in achieving a more youthful look. Other regions of the face and body that benefit from autologous fat grafting include the chin, submental, earlobe, hand, and perioral regions.^6,19^ The fat compartments of each region have been described and are augmented if there is any volume loss. Most of these regions in the face are centrally located and are not adequately treated by face-lift alone.

Other ancillary procedures include a lip-lift procedure to shorten an upper lip with labral excess. Lastly, the texture or skin quality is not addressed with a “lift-and-fill” face-lift,^20^ hence we recommend useful adjuncts such as laser resurfacing and chemical peels. The key in the treatment of facial aging is formulating an individualized plan for each patient based on a thorough facial analysis.^21,22^

## GENERAL TECHNIQUE OF AUTOLOGOUS FAT HARVEST AND TRANSFER

For autologous fat transfer and preparation, adipose tissue is harvested from the medial thighs using a 10-mL syringe with a 14-gauge cannula. The thigh tends to have less pain compared to other donor sites. No wetting or infiltration solution is used. The harvested lipoaspirate is centrifuged at 2,250 rpm for 1 minute followed by removal of the infranatant and supernatant to isolate concentrated fat. One to two milliliters of this concentrate is transferred at a time to each facial fat compartment requiring volumization, which was injected using a 16-gauge blunt cannula and a 1-mL syringe. The fat is injected with anterograde and retrograde motions of the syringe at a low pressure.

Fractionated fat,^23^ or autologous fat that is fractionated by emulsification, is prepared with a few additional steps. The concentrated fat that is obtained is transferred between two 10-mL syringes approximately 50 times to emulsify the harvested autologous fat. This process fragments the fat without compromising viability. The fractionated fat is grafted using 2-mm blunt cannulas into periorbital fat compartments.

## BLENDING LOWER EYELID-CHEEK JUNCTION

The harsh transition of the medial sub-orbicularis oculi fat pad and the malar fat pad creates the appearance of an aging lid-cheek injuction.^24^ The periorbital and malar cheek fat pads atrophy earlier in life compared with the neighboring fat compartments.^12^ Loss of volume and weakening of the retaining ligaments cause the tell tale signs of aging of the midface termed the V-deformity. This deformity is closely associated with the presence of a tear trough deformity and nasojugal groove. To address these deformities, an understanding of the major fat compartments of the midface is key.

One major fat compartment that is not well addressed by traditional face-lift is the nasolobial fat compartment. This compartment is located anterior to the medial cheek fat compartment and is bordered superiorly by the orbicular retaining ligament.^13^ The major superficial fat compartments of the cheek are the medial, middle, and lateral temporal compartments. The medial compartment is lateral to the nasolabial fat compartment and inferior to the orbicular retaining ligament.^13^ The middle and lateral fat compartments are superifical to the parotid gland. The orbital fat compartments have 3 components: superior, inferior, and lateral.^25^

The deep medial cheek fat is deep to the superficial muscular aponeurotic system (SMAS) and the superficial cheek fat compartments.^14^ This compartment is anterior to the maxilla and Ristow’s space. It has a medial and lateral portion which are both medial to the zygomaticus major muscle. Autologous fat grafting to the deep cheek compartment addresses the V-deformity and prominent nasolabial folds to achieve a more youthful appearance. The nasolabial fat compartments and the superficial middle and lateral cheek compartments can be filled as needed to improve the final contour.^13^ These are the most important fat compartments to address when blending the lid-cheek junction. Fat is injected into these compartments using the alar base and lateral cheek as entry points. A 2-mm blunt cannula is inserted to inject the fat into the deep medial cheek fat and lateral superficial cheek compartments.

The forehead and temporal fat compartments contain a central compartment with a middle compartment lateral to it.^13^ The lateral temporal-cheek compartment is lateral to the middle compartment. This compartment is contiguous with the lateral superficial compartment of the cheek. In patients with hollowing of these regions, 1–2 mL of autologous fat can be injected to volumize these regions.

**Fig. 2.**
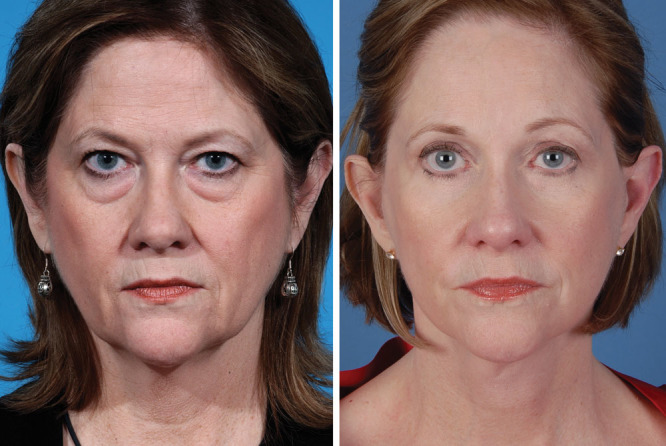
This patient underwent a lift-and-fill face-lift along with periorbital rejuvenation with a 6-step blepharoplasty. Autologous fat grafting using fractionated fat along with the release of the tear trough ligament helped blend the lid-cheek junction and efface the preoperative tear trough deformity. The patient has a more youthful contour in her midface.

To augment the periorbium,^26^ fractionated fat is grafted using fine 2-mm blunt cannulas into the cheek-orbital rim junction to disrupt orbital malar ligament below the orbicularis muscle.^23,27^ An incision is made lateral to the orbital rim and 1–2 mL is also injected deep to the muscle in the lower eyelid. Fifty percentage of overcorrection is the goal. The maneuver augments the lower eyelid and disrupts the malar ligament which contributes to the presence of a tear trough deformity^28^ (**SDC 1**; **see** video, Supplemental Digital Content 1, which displays fat grafting to the periorbium, http://links.lww.com/PRSGO/B86). There is slightly more bruising and edema with grafting to the perioribium but we have shown fractionated fat to contain viable fat cells for transfer.^23^ In our experience, we have seen improved contour and skin texture (Fig. 2).

**Video graphic 1.**
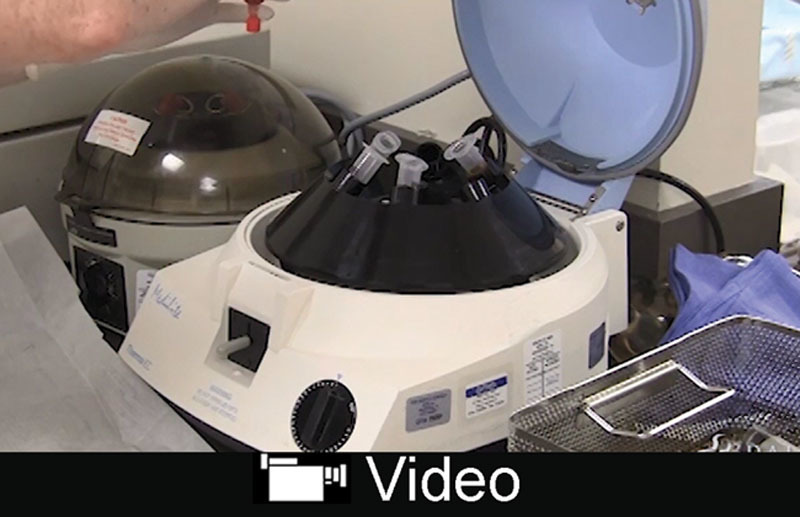
See video, Supplemental Digital Content 1, which displays fat grafting to the periorbium. Fat harvesting technique is shown along with how fractionated fat is made and injected. Fractionated fat is used to augment and fill the periorbium in 1-mL aliquots in an anterograde and retrograde fashion. This video is available in the “Related Videos” section of the Full-Text article at PRSGlobalOpen.com or at http://links.lww.com/PRSGO/B86.

## CHIN AUGMENTATION

**Fig. 3.**
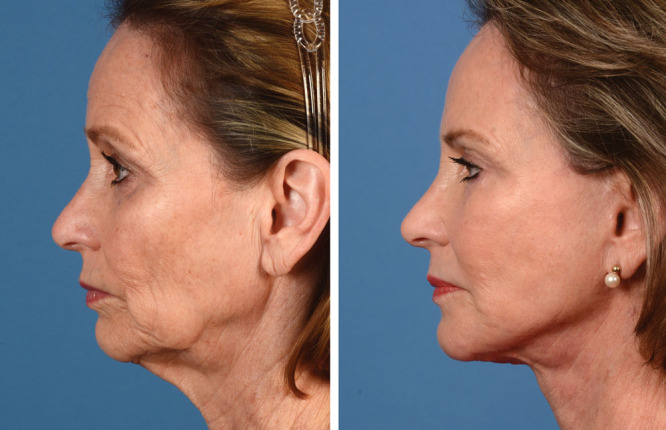
This patient who underwent a lift-and-fill face-lift also had chin augmentation with autologous fat. The face-lift helped improve her cervicomental angle and eliminate jowling that was present preoperatively. In addition, her microgenia was corrected with facial fat augmentation. Published with permission from *Plast. Reconstr. Surg.* 2018;142:921–925.

The chin is another region not addressed by a traditional face-lift but can show signs of aging.^29,30^ Rhytids and deflation of fat compartments can occur in this region. Microgenia is assessed based on a line drawn from the upper lip to the menton. Many of the deformities of the chin associated with aging are best addressed with facial fat augmentation as opposed to placement of an implant.^30^ Fat grafting can address lateral chin hollowing, microgenia, labiomental sulcus, and a midline cleft.^31^ The access sites are the midline to address a bifid chin; 2 lateral sites for lateral chin hollowing; and 1 for the labiomental crease. Fat concentrate of 1–2 mL is placed in each region and overcorrected 50% in women and 100% in men^30^ (Fig. 3; **SDC 2**, **see** video, Supplemental Digital Content 2, which displays chin augmentation with autologous fat grafting, http://links.lww.com/PRSGO/B87).

**Video graphic 2.**
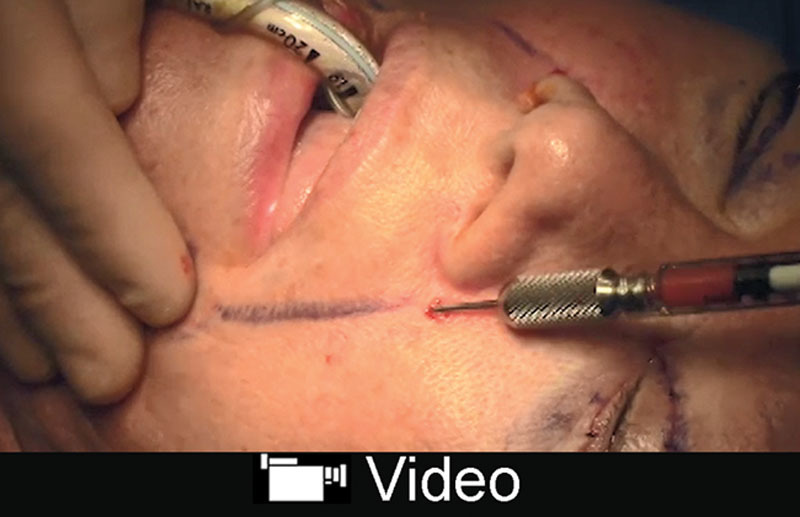
See video, Supplemental Digital Content 2, which displays chin augmentation with autologous fat grafting. Autologous fat grafting can help address microgenia to augment a retruded chin. Injections are performed into the lateral compartments of the chin and the central compartment. Labiomental sulcus is augmented if needed. This video is available in the “Related Videos” section of the Full-Text article at PRSGlobalOpen.com or at http://links.lww.com/PRSGO/B87.

## EARLOBE REJUVENATION

**Fig. 4.**
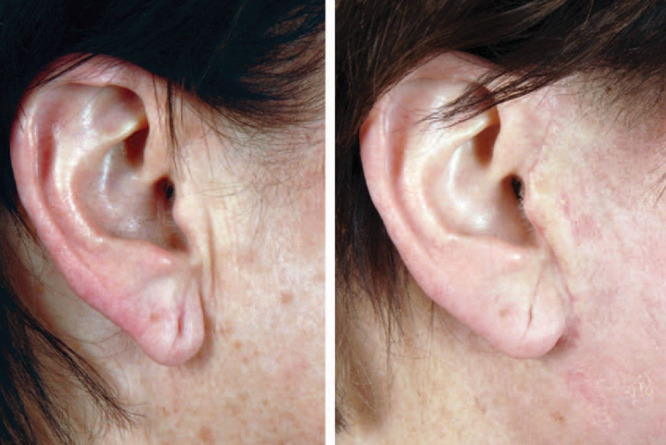
Preoperative (a) and postoperative (b) photographs of a woman who underwent rhytidectomy with autologous fat transfer to her lobule. The patient had a deflated ear lobule preoperatively and after autologous fat transfer, the lobule is wider and more youthful. Published with permission from *Plast Reconstr Surgery Glob Open.* 2016;4:e597.

The ear lobule is a fibrofatty structure without cartilage which atrophies with age. A youthful ear lobule is 1.5–2 cm in length. The aesthetic ideal of a lobule is voluminous with a round projection. An aging lobule seems thin with a flat appearance.^32^ The free caudal segment of the lobule is unaffected by a face-lift. We described the technique of autologous fat grafting to the lobule by injecting ~1–3 mL of autologous fat onto the anterior surface of the lobule at the same time as a face-lift^33^ (**SDC 3, see** video, Supplemental Digital Content 3, which displays autologous fat grafting to face, http://links.lww.com/PRSGO/B88.) Patients do not wear earrings for 2 weeks after surgery. The results are maintained for at least 1 year postoperatively (Fig. 4).

**Video graphic 3.**
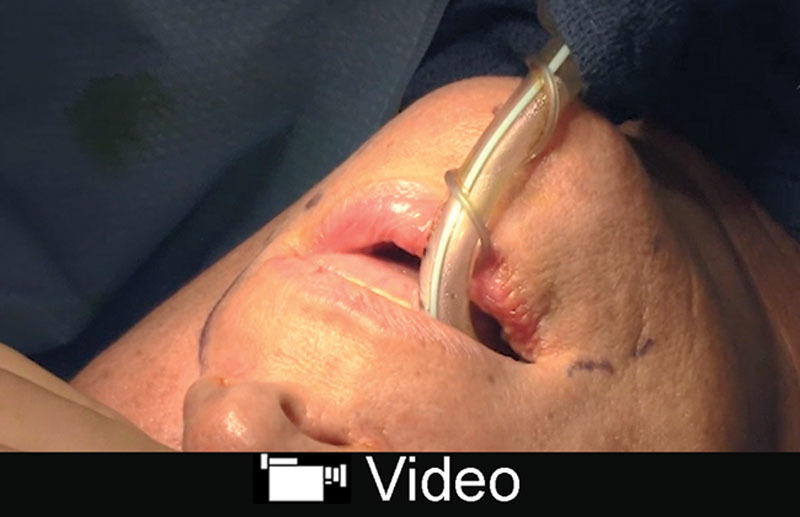
See video, Supplemental Digital Content 3, which displays autologous fat grafting to face. The perioral region and fat compartments neighboring the lateral commissure can be augmented with autologous fat. The lobule contains fibrofatty tissue that can atrophy with age and this area can be fat grafted to augment and improve the appearance of the lobule. This video is available in the “Related Videos” section of the Full-Text article at PRSGlobalOpen.com or at http://links.lww.com/PRSGO/B88.

## HAND REJUVENATION

**Fig. 5.**
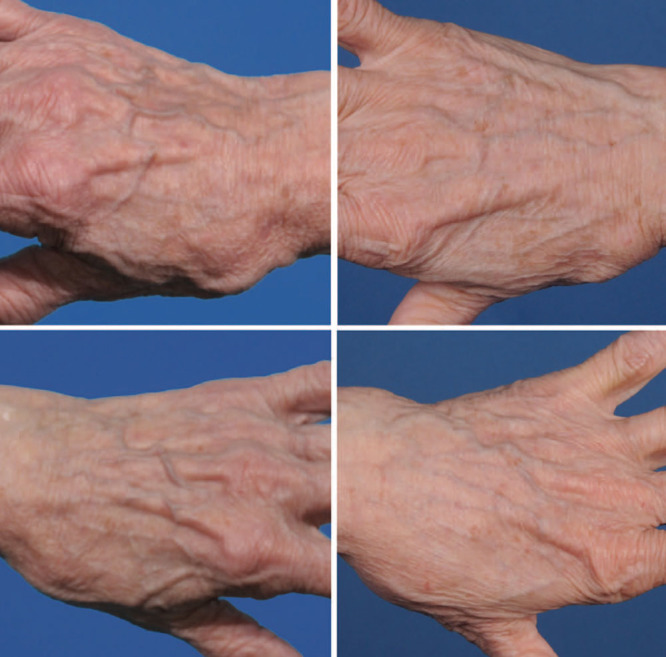
This 77-year-old patient underwent facial rhytidectomy and hand rejuvenation with autologous fat grafting. Twenty milliliters of fat was grafted to each hand. Seven months postoperatively, the patient obtained desired aesthetic results, with restoration of fullness, contour, and decreased prominence of dorsal hand structures. A, Preoperative right hand. B, Seven-month postoperative right hand. C, Preoperative left hand. D, Seven-month postoperative left hand. Published with permission from *Plast Reconstr Surg.* 2015;136:1175–1179.

Aging hands can reveal a person’s age but soft tissue volume loss can also be addressed by autologous fat grafting.^34–36^ The dorsum of the hand is split into 3 distinct fat compartments which layer this region. Autologous fat grafting to the superficial and intermediate fat compartments is performed. Stab incisions are made between the third and fourth metacarpophalangeal (MCP) joints. A 10-mL syringe with a 14-gauge blunt cannula is used to inject ~15 mL of fat into the distal two thirds of the dorsal hand. A separate stab incision is made between the first and the second MCP joints to inject 10-mL of fat into the proximal one third of the dorsal hand and the dorsal thenar eminence (**SDC 4, see** video, Supplemental Digital Content 4, which displays autologous fat transfer to the hand, http://links.lww.com/PRSGO/B89). The filling mitigates the appearance of the underlying soft tissue structures. After the procedure, the hand is dressed with a light compressive garment and removed in 24 hours (Fig. 5).

**Video graphic 4.**
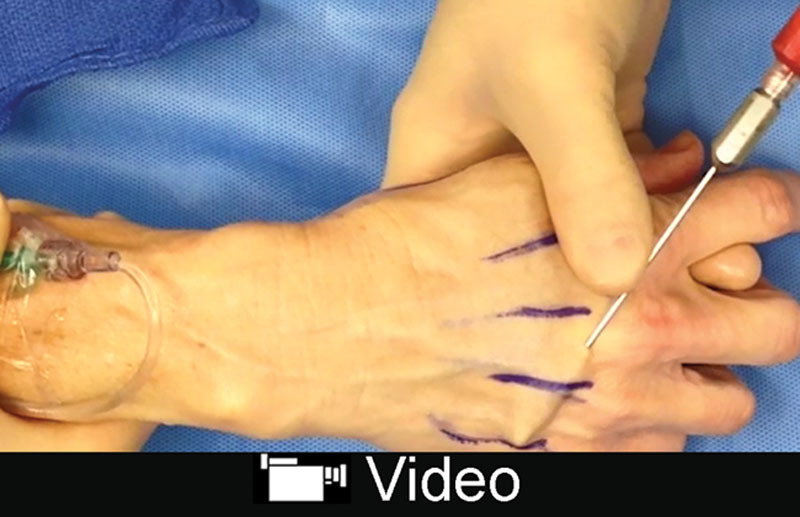
See video, Supplemental Digital Content 4, which displays autologous fat transfer to the hand. The distal two thirds of the hand are augmented first followed by the proximal one third of the dorsal hand. The grafted fat is smoothed throughout the area to prevent any formation of lumps or deformities. This video is available in the “Related Videos” section of the Full-Text article at PRSGlobalOpen.com or at http://links.lww.com/PRSGO/B89. Published with permission from *Plast Reconstr Surg.* 2015;136:1175–1179.

## PERIORAL REJUVENATION

With aging in the perioral region, there are prominent rhytids and volume loss in the fat compartments which are not addressed with a traditional face-lift.^29,37^ Understanding of the facial anatomy followed by volume restoration of this region creates a more harmonious result. The aesthetic ideal of the perioral region has a distinct vermilion-cutaneous border with a prominent pout and well-defined philtral columns. There is typically 2–3 mm of upper incisor show in repose. There is a natural curve seen on lateral view from the nostril sill to the vermilion border that is lost with age. The upper lip aesthetic unit consisting of the dry vermilion and the ergotrid which is bordered by the vermilion border, nasal base, and nasolabial folds.^38^ The lip is usually 25% of the height of labrum. The upper lip volume is 75%–80% of the lower lip volume. The aging process causes the vermilion to become thin and fine vertical rhytids appear as the facial volume decreases and descends.^38^

**Fig. 6.**
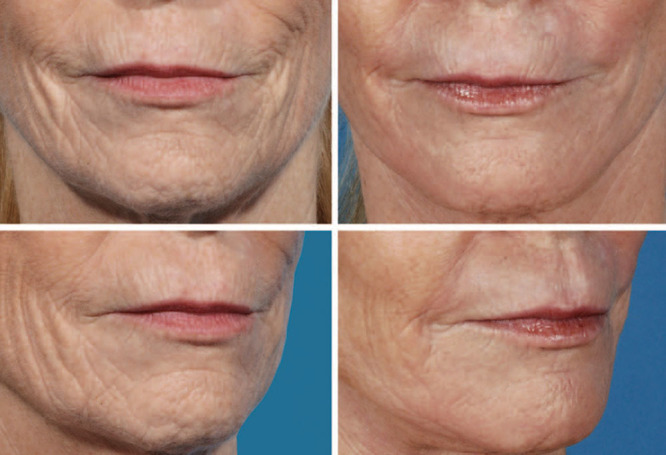
Preoperative (A) and postoperative (B) photographs of a woman who underwent rhytidectomy with perioral rejuvenation using autologous fat. The perioral regions seem more youthful with more fullness after the treatment. Published with permission from *Plast Reconstr Surg.* 2015;136:301e–309e.

Lip resurfacing using dermabrasion, light-based therapies, or chemical pills can address fine perioral rhytids. Perioral fat compartments can be filled without autologous fat. There are superficial and deep facial fat compartments in this region and the deeper compartments are deep to the orbicularis muscle^14,29^ (Fig. 6). Hyaluronic acid–based filler is recommended for volumization of the upper and lower lip white roll. Autologous fat grafting to the white roll can create an asymmetric result. A loss of upper or lower lip volume is best addressed with volumization and filling.

**Fig. 7.**
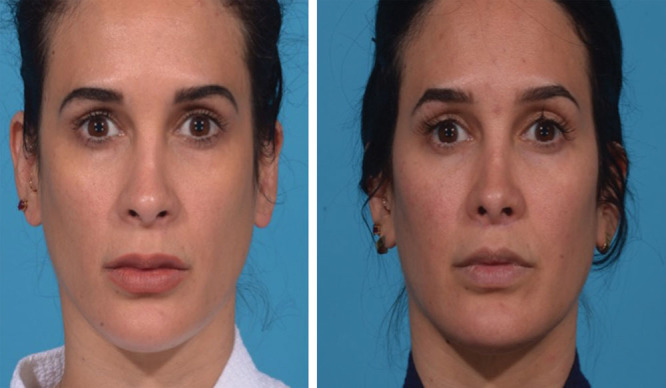
This young patient underwent an upper lip-lift to treat a long, elongated lip. In her postoperative photo, her scar is barely noticeable. She has an improved contour and greater youthful convexity of her philtrum and upper lip.

Lip-lift is an adjunct used to decrease lip ptosis, increase eversion of the vermilion, and increase incisal show. This operation primarily addresses deformities associated with the height of the labrum. The operation involves excising skin and soft tissue just inferior to the nostril sill at the uppermost aspect of the upper lip^38^ (Fig. 7; **SDC 5, see** video, Supplemental Digital Content 5, which displays upper lip-lift, http://links.lww.com/PRSGO/B90).

**Video graphic 5.**
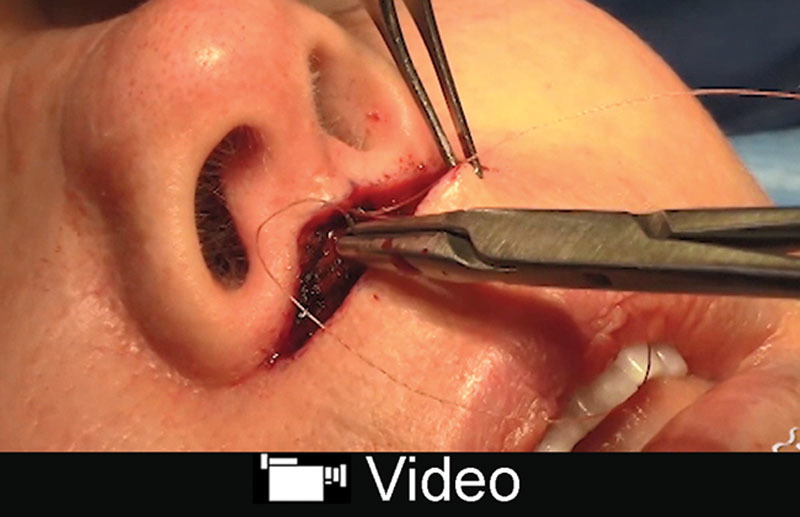
See video, Supplemental Digital Content 5, which displays upper lip-lift. This procedure serves as an excellent adjunct for the aging face because it can help address a long upper lip. The scar will camouflage well with the surrounding tissue. This video is available in the “Related Videos” section of the Full-Text article at PRSGlobalOpen.com or at http://links.lww.com/PRSGO/B90.

## LASER RESURFACING

Face-lifts with laser resurfacing has shown to be a safe and effective technique for rejuvenation of the face.^39–41^ Laser resurfacing addresses dyschromia, textural changes, and rhytids. The Er:YAG laser provides ablative resurfacing with a lower risk profile and faster healing compared to carbon dioxide lasers. It is only used on patients who are Fitzpatrick type I or II to prevent any risk of posttreatment pigmentary changes. Depending on the patient, laser resurfacing can be used for the (1) perioral region alone, (2) central face, or (3) full face. Central face includes the perioral area, medial cheeks, forehead, glabella, and lower eyelid.

**Fig. 8.**
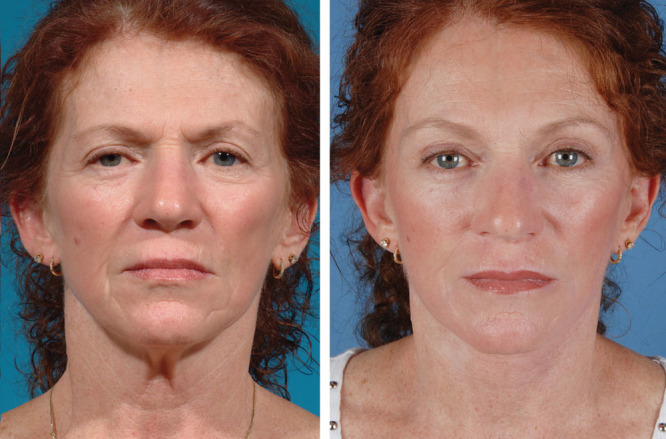
This patient underwent facial rhytidectomy along with full facial resurfacing with an erbium dual-mode laser. Her texture and skin quality are greatly improved with both treatments and she seems considerably younger in her postoperative photo. Notice that the perioral rhytids improved creating a more full, youthful look.

Patients were treated with tretinoin for a 4- to 6-week period before the laser resurfacing treatment. Lymphatic massage is begun 2 weeks before the procedure. Patients receive acyclovir 500 mg 4 times a day for 7 days before the procedure. After the procedure, patients begin a methylprednisolone dose pack. The settings for the laser are 20–25 J, 80–100 µm ablation, and 50% overlap with at least 2 passes in nonundermined areas. Spot passes are performed in the perioral region with settings of 5–25 J (**SDC 6, see** video, Supplemental Digital Content 6, which displays full facial resurfacing with an erbium dual-mode laser, http://links.lww.com/PRSGO/B91). Similar settings are used for the central face and full face resurfacing. Flexzan dressings are used postoperatively to dress the treated areas and removed in 2–4 days. Moisturizing creams are applied 2–3 times daily to keep the skin hydrated. With this treatment regimen, there were no cases of skin necrosis or sloughing in our review of fully undermined face-lifts flaps treated with ablative laser resurfacing^40^ (Fig. 8). The overall complication rate was 3.8%. In these patients who underwent ablative resurfacing at the time of face-lift, the 3 complications were as follows: hyperpigmentation, prolonged erythema, and delayed wound healing at the tragus.

**Video graphic 6.**
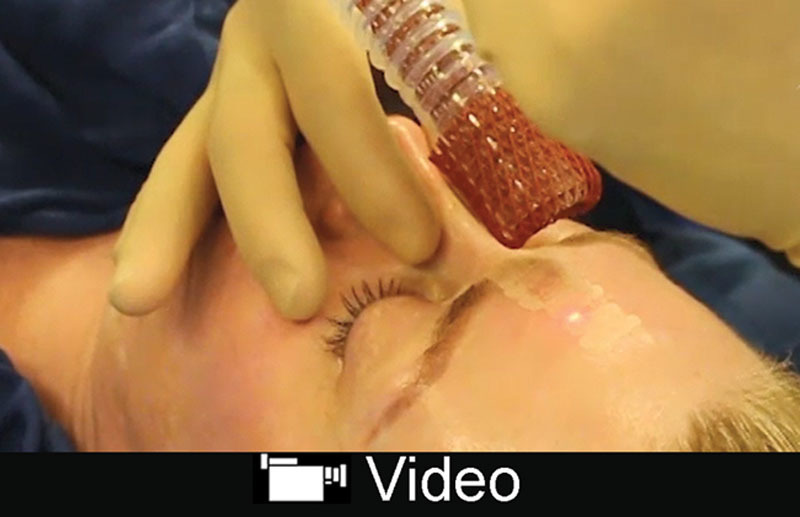
See video, Supplemental Digital Content 6, which displays full facial resurfacing with an erbium dual-mode laser. Full facial resurfacing with a laser is an excellent ancillary procedure to address dyschromias and rhytids. The senior author performs laser resurfacing in an ablative mode to various anatomic regions of the face. This video is available in the “Related Videos” section of the Full-Text article at PRSGlobalOpen.com or at http://links.lww.com/PRSGO/B91.

## CHEMICAL PEELS

As an adjunct to a lift-and-fill face-lift,^20^ chemical peels using trichloroacetic acid and Jessner’s peel provided a medium-depth peel which are effective in addressing dyschromias and rhytids.^42^ This peel’s depth of penetration is the reticular dermis. All patients undergo a prepeel treatment regimen consisting of topical tretinoin, hydroquinone, and an alpha hydroxyl acid that are all applied twice a day for a 5-week period until 1 week before the peel. A week prior the peel, patients apply moisturizer and sunscreen to protect their skin and keep it well hydrated in preparation for the procedure. In patients with a history of herpetic lesions, prophylactic treatment is begun 2 days before the peel and continued for 5 days afterward.

The peel is begun by applying 70% ethyl alcohol which cleanses the skin and is followed by acetone which degreases the skin. After the skin is allowed to dry, Jessner’s solution (100 mL of 95% ethanol, 14 g resorcinol, 14 g salicylic acid, and 14 mL lactic acid) is applied to act as an exfoliant and it penetrates to the basal epidermis. The 35% trichloroacetic acid (TCA) solution is then applied using 2 × 2 gauze pads (**SDC 7, see** video, Supplemental Digital Content 7, which displays chemical peel with TCA peel, http://links.lww.com/PRSGO/B92). The solution is soaked in gauze pads and light passes are made over the areas requiring treatment until a light frost develops. A pink-white hue demonstrates penetrance into the papillary dermis, whereas a uniform white frost indicates penetration in the reticular dermis. The frost can last 30–40 minutes after the peel is completed. In the posttreatment recovery period, the skin will slough over a 7- to 10-day period. Patients are instructed to wash the skin gently and apply petrolatum-based ointments or creams. Excessive moisture prevents peeling and extreme dryness delays re-epithelialization of the skin, hence a balance must be achieved. With this regimen, we have reported a very low complication rate.^42^ Jessner’s solution aids in penetration and evenness of the TCA peel, which helps collagen remodeling and improvement in the appearance of the skin.

**Video graphic 7.**
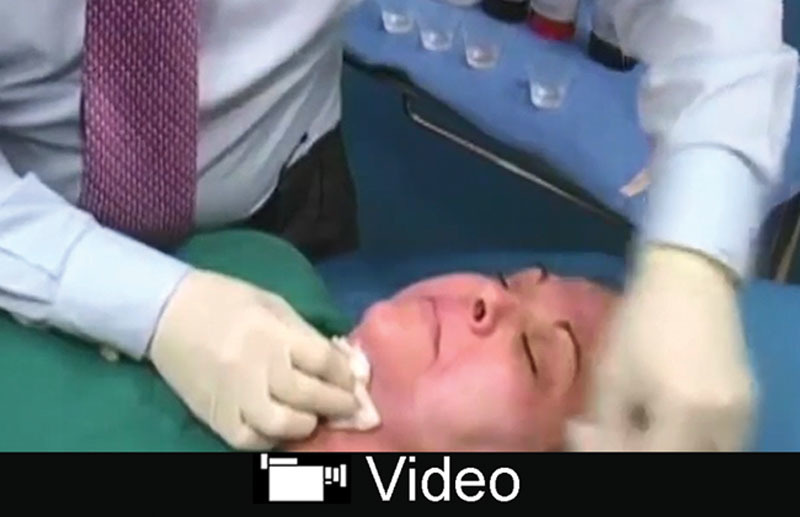
See video, Supplemental Digital Content 7, which displays chemical peel with TCA peel. First, alcohol and acetone are applied to the areas to be treated. Then a Jessner’s peel is performed to ensure a more uniform treatment with the TCA peel. A TCA medium-depth peel is applied to rejuvenate the face. The endpoint is to achieve a white frost. This video is available in the “Related Videos” section of the Full-Text article at PRSGlobalOpen.com or at http://links.lww.com/PRSGO/B92. Published with permission from *Plast Reconstr Surg.* 2009;124:965–966.

## CONCLUSIONS

Although a traditional face-lift will rejuvenate the face, it primarily addresses lateral facial skin laxity and when done alone, this procedure lacks the ability to rejuvenate and harmonize all aging regions of the face. With the advent of newer autologous fat grafting techniques and detailed knowledge of the soft tissue anatomy of the face, ancillary procedures in facial rejuvenation improve on the results of a face-lift by filling and volumizing regions of the face that have undergone fat atrophy and soft tissue deflation. In addition, ancillary procedures such as lasers and peels can improve the texture and appearance of the skin. These procedures are integral in the modern face-lift.

## ACKNOWLEDGMENTS

The authors would like to thank Dr. Paul Afrooz and Martha Aceves for their assistance in video production and video editing.

## Supplementary Material
